# Ag_2_CO_3_ Decorating BiOCOOH Microspheres with Enhanced Full-Spectrum Photocatalytic Activity for the Degradation of Toxic Pollutants

**DOI:** 10.3390/nano8110914

**Published:** 2018-11-07

**Authors:** Shijie Li, Liuye Mo, Yanping Liu, Huiqiu Zhang, Yaming Ge, Yingtang Zhou

**Affiliations:** 1 Key Laboratory of Key Technical Factors in Zhejiang Seafood Health Hazards, Institute of Innovation & Application, Zhejiang Ocean University, Zhoushan 316022, China; liuyemo@zjou.edu.cn (L.M.); zhanghuiqiu2006@163.com (H.Z.); geyaming@126.com (Y.G.); zhouyingtang@zjou.edu.cn (Y.Z.); 2 Department of Environmental Engineering, Zhejiang Ocean University, Zhoushan 316022, China

**Keywords:** Ag_2_CO_3_/BiOCOOH, heterojunction, photocatalysis, pollutant removal, full-spectrum

## Abstract

The development of excellent full-spectrum photocatalysts is of vital significance to its practical application in environmental remediation. Herein, flower-like Ag_2_CO_3_/BiOCOOH type I heterostructures were prepared via a facile method and exhibited powerful photocatalytic activity by removing various toxic pollutants (rhodamine B, methyl blue, and tetracycline hydrochloride) under simulated sunlight irradiation. The boosted photocatalytic performance is attributed to the expanded range of the absorption spectrum and alleviated separation rate of the photo-induced electrons and holes. The photoluminescence spectra and trapping experiment were applied to clarify the photocatalytic reaction mechanism of Ag_2_CO_3_/BiOCOOH. The holes and •O_2_^−^ were detected as the dominant reactive species involved in pollutant degradation. This work provides a novel full-spectrum-driven photocatalyst of Ag_2_CO_3_/BiOCOOH, which could effectively degrade toxic pollutants under simulated sunlight.

## 1. Introduction

Over the past few decades, toxic contaminants (e.g., industrial dyes and antibiotics) in water discharged by chemical industries have caused serious environmental pollution. However, conventional water treatment cannot efficiently degrade the contaminants. The application of photocatalytic technology in treating environmental pollution is regarded as an eco-friendly and sustainable technology to afford organic contaminant degradation [1,2,3]. The exploration of a novel full-spectrum-driven photocatalyst that can make the best use of sunlight has an important value in its practical application. Recently, great attention has been paid to Bi-based photocatalysts [4,5,6,7,8,9,10,11]. Among them, BiOCOOH is a kind of layered Bi-based oxide, built from [Bi_2_O_2_]^2+^ fluorite-like layers intercalating by formic acid [12,13]. As an active ultraviolet-light-driven (ULD) photocatalyst, BiOCOOH possesses some advantages such as high chemical stability, a strong photo-redox driving force, and decent activity. However, the wide band gap (*E*_g_ = 3.4 eV) of BiOCOOH severely restrains its practical application. Actually, the combination of wide band gap semiconductors with narrow band gap compounds can remarkably enhance the photocatalytic performance, due to expanded sunlight absorption and the accelerated separation of charge carriers [14,15,16,17,18]. For instance, Aguirre et al. have recently employed Cu_2_O coated by TiO_2_ for improving photocatalytic stability and performance [16]. As a result, BiOCOOH was coupled with Ag_2_O [19], CNT [20], RGO [21], C_3_N_4_ [22], BiOI [23], CQDs [24] and so on. Of note, the further exploration of fascinating full-spectrum-driven BiOCOOH-based heterojunction photocatalysts is still challenging.

Ag_2_CO_3_, as a highly active visible-light-driven photocatalyst, has been recognized as a good photosensitizer, since its narrow band gap (~2.17 V) is advantageous for sunlight absorption [25,26,27]. Hence, the design of Ag_2_CO_3_-based heterojunction photocatalysts (e.g., Ag_2_CO_3_/Ag_2_O [28], Ag_2_CO_3_/Ag_2_S [29], Ag_2_CO_3_/Bi_2_MoO_6_ [8,30], Ag_2_CO_3_/BiOCl [31], Ag_2_CO_3_/Ag/WO_3_ [32] and Ag_2_CO_3_/AgBr/ZnO [33]) to improve the visible-light photocatalytic performance has been achieved by scholars. However, to the best of our knowledge, no Ag_2_CO_3_/BiOCOOH heterojunctions have been studied. Hence, it inspires us to combine BiOCOOH with Ag_2_CO_3_ for obtaining novel Ag_2_CO_3_/BiOCOOH heterojunctions with outstanding sunlight-driven photocatalytic performance.

In this study, aiming at the construction of highly active sunlight-driven photocatalysts, flower-like Ag_2_CO_3_/BiOCOOH heterojunctions were prepared via a simple precipitation approach. Under simulated sunlight irradiation, the photocatalytic property of Ag_2_CO_3_/BiOCOOH in the removal of various toxic pollutants (rhodamine B (RhB), methyl blue (MB), and tetracycline hydrochloride (TC)) was evaluated. In addition, the photocatalytic mechanism of Ag_2_CO_3_/BiOCOOH was also illustrated based on the experimental results.

## 2. Materials and Methods

### 2.1. Chemicals

Bi(NO_3_)_3_•5H_2_O, rhodamine B (RhB), NaHCO_3_, NH_3_•H_2_O, glycerol, methyl blue (MB), ammonium oxalate (AO), tetracycline hydrochloride (TC), *N*,*N*-dimethylformamide (DMF), iso-propanol (IPA), and *p*-benzoquinone (BQ) were purchased from the Chinese Chemical Reagent factory and used as was received without further treatment.

### 2.2. Synthesis of Catalysts

BiOCOOH microspheres were prepared solvothermally. Typically, Bi(NO_3_)_3_•5H_2_O (4 mmol) is dissolved in 50 mL of glycerol under vigorous stirring. After dissolution, 20 mL of DMF and 10 mL of H_2_O were added into the above solution and stirred for 0.5 h. The obtained solution was poured into an autoclave with a volume of 100 mL and solvothermally treated at 160 °C for 24 h. After the reaction ended, the products collected from the suspension were washed with deionized water and ethanol four times, and finally transferred into an oven to get dried at 60 °C for 15 h.

Flower-like Ag_2_CO_3_/BiOCOOH heterojunctions were fabricated by chemical deposition. Firstly, a certain amount of BiOCOOH was evenly dispersed in 50 mL of deionized water during magnetic stirring. Subsequently, 1 mmol AgNO_3_ was dissolved in the above system during magnetic stirring. Then, 10 mL of NaHCO_3_ solution (0.05 M) was dropped into the above system slowly and stirred for another 2 h in darkness. Following this, the precipitant was washed thoroughly, and dried at 60 °C for 6 h. By changing the addition amount of BiOCOOH, the Ag_2_CO_3_/BiOCOOH heterojunctions with different mass ratios (0.3/1, 0.5/1, 1/1 and 1.5/1) were prepared and labelled as ACO/BOCH-30, ACO/BOCH-50, ACO/BOCH-100, and ACO/BOCH-150, respectively.

### 2.3. Characterization

X-ray powder diffraction (XRD) was conducted on a Bruker D8 Advance diffractometer (Cu Kα = 1.5406 Å) with a scanned range of 2θ from 10° to 80° (Karlsruhe, Germany). Scanning electron microscope (SEM, Hitachi S–4800, accelerating voltage = 10 kV, Tokyo, Japan), energy dispersive X-ray spectroscopy (EDX) (Berlin, Germany), and transmission electron microscope (TEM, JEM–2010F, Tokyo, Japan) were employed to study the morphologies and compositions of the catalysts. The UV-vis diffused reflectance spectra (DRS) were collected on a spectrophotometer (Shimadzu UV-2600, Tokyo, Japan). Room-temperature photoluminescence (PL) spectra were detected by using a Hitachi F-7000 fluorescence spectrophotometer (Tokyo, Japan).

### 2.4. Photocatalytic Performance Tests

The photo-degradation of rhodamine B (RhB), methyl blue (MB) or tetracycline hydrochloride (TC) under simulated sunlight was performed to assess the activity of catalysts. Briefly, 30 mg of catalyst was dispersed evenly in 100 mL of RhB (10 mg/L), MB (10 mg/L), or TC (20 mg/L) solution while magnetically stirring for 30 min in the dark to establish an adsorption-desorption equilibrium [34,35]. The reaction system was maintained at room temperature by using circulating water. Subsequently, the above system was illuminated by a 300 W xenon lamp with an average light intensity of 4.56 KW/m^2^. During illumination, approximately 2 mL of the suspensions were sampled at certain intervals, and then centrifuged to get the supernatant solutions. The concentrations of solutions were quantified by using a UV-2600 spectrophotometer. Total organic carbon (TOC) experiments were carried out by the decomposition of RhB (50 mg/L, 200 mL) solution over 200 mg of ACO/BOCH-100 under simulated sunlight. The reaction solution collected at given intervals was detected by a Shimadzu TOC-VCPH TOC analyzer. To test the stability and reusability of ACO/BOCH-100, six successive runs for RhB degradation were performed, and each run lasted 30 min. After one cycle of photocatalytic reaction, the catalysts recycled by centrifugation and were washed thoroughly with deionized water and dried at 70 °C overnight. Subsequently, the dried catalysts were used to degrade the RhB solution again. During the recycling process, about 4.7 mg of the catalyst was lost.

## 3. Results and Discussion

### 3.1. Characterization

**Figure 1:**
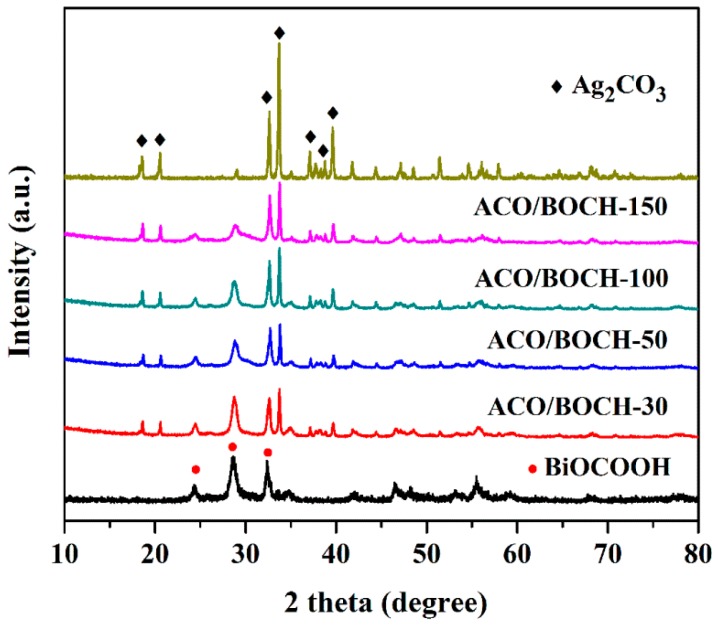
XRD patterns of pure BiOCOOH, Ag_2_CO_3_, and Ag_2_CO_3_/BiOCOOH heterojunctions (ACO/BOCH-30, ACO/BOCH-50, ACO/BOCH-100, and ACO/BOCH-150).

A series of Ag_2_CO_3_/BiOCOOH heterojunctions with various mass ratios (0.3/1, 0.5/1, 1/1, and 1.5/1) were fabricated and symbolized as ACO/BOCH-30, ACO/BOCH-50, ACO/BOCH-100, and ACO/BOCH-150, respectively. The crystallographic structures of as-prepared BiOCOOH, Ag_2_CO_3_, and Ag_2_CO_3_/BiOCOOH heterojunctions (ACO/BOCH-30, ACO/BOCH-50, ACO/BOCH-100, and ACO/BOCH-150) were studied by XRD (Figure 1). The diffraction peaks of BiOCOOH and Ag_2_CO_3_ coincided well with those of tetragonal BiOCOOH (JCPDS 35-0939) [13] and monoclinic Ag_2_CO_3_ (JCPDS 26-0399) [25,26], respectively. The XRD patterns of Ag_2_CO_3_/BiOCOOH heterojunctions display that both Ag_2_CO_3_ and BiOCOOH phases were detected in these heterojunctions, confirming the co-existence of Ag_2_CO_3_ and BiOCOOH in these as-prepared heterojunctions. Additionally, no trace of any impurity phase was detected.

**Figure 2:**
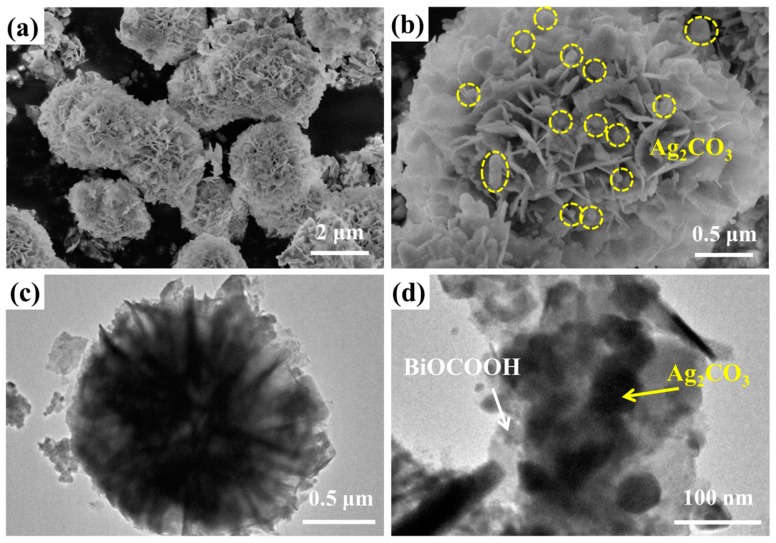
(**a**,**b**) SEM and (**c**,**d**) TEM images of ACO/BOCH-100.

**Figure 3:**
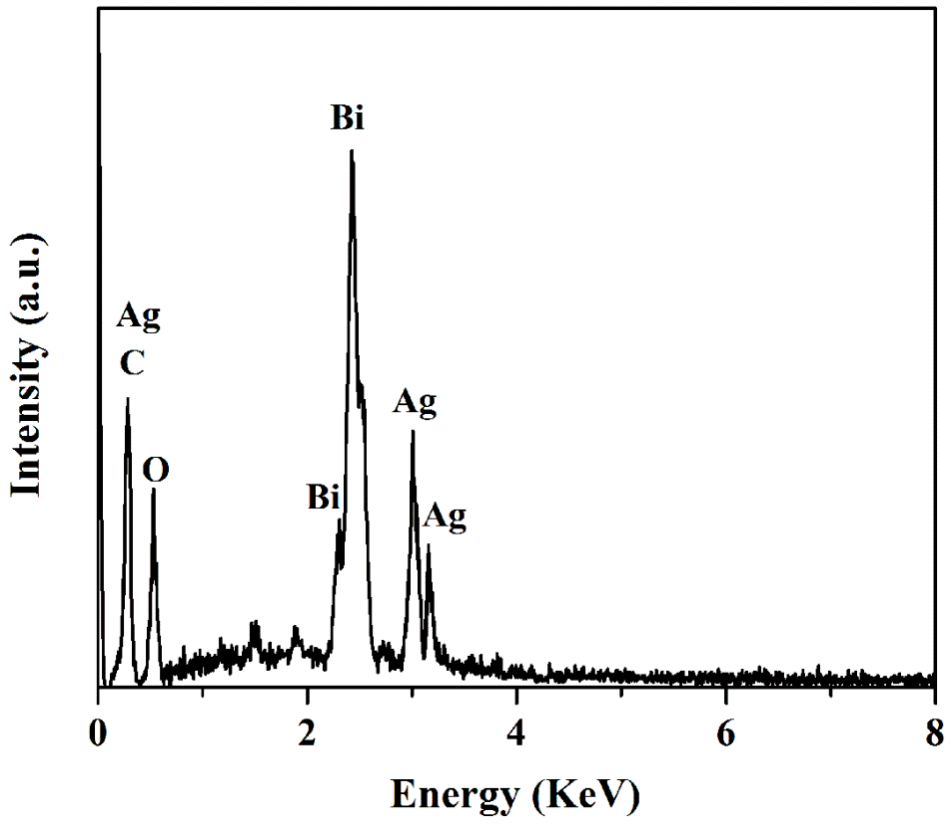
EDS spectra of ACO/BOCH-100.

The morphologies of BiOCOOH and the Ag_2_CO_3_/BiOCOOH heterojunction (ACO/BOCH-100) were visualized by SEM and TEM (Figure 2 and Figure S1). Pure BiOCOOH presented a flower-like structure (diameter: 1.5-2.5 μm), which was composed of numerous nanoplates (Figure S1). After its combination with Ag_2_CO_3_, the obtained Ag_2_CO_3_/BiOCOOH (ACO/BOCH-100) displayed a similar morphology to the BiOCOOH (Figure 2a). Further magnification of the image as presented in Figure 2b revealed that Ag_2_CO_3_ nanoparticles (size: 30-150 nm) were anchored onto the BiOCOOH microsphere. Further observation through TEM imaging (Figure 2c,d) confirmed that ACO/BOCH-100 was a flower-like architecture decorated by Ag_2_CO_3_ nanoparticles, verifying the successful fabrication of Ag_2_CO_3_/BiOCOOH with an intimately contacted interface. Furthermore, as shown in the energy dispersive spectroscopy (EDS) spectra, Ag, C, O, and Bi in ACO/BOCH-100 could be detected, testifying that the sample was Ag_2_CO_3_/BiOCOOH (Figure 3).

The surface texturing of BiOCOOH and ACO/BOCH-100 were analyzed by the N_2_ adsorption–desorption isotherms (Figure S2). The BET specific surface areas of BiOCOOH and ACO/BOCH-100 were 26.2 and 22.7 m^−2^ g^−1^, respectively. The BJH pore-size distributions revealed the presence of nanopores with the main pore size of 31 nm in BiOCOOH and ACO/BOCH-100 (the inset of Figure S2).

**Figure 4:**
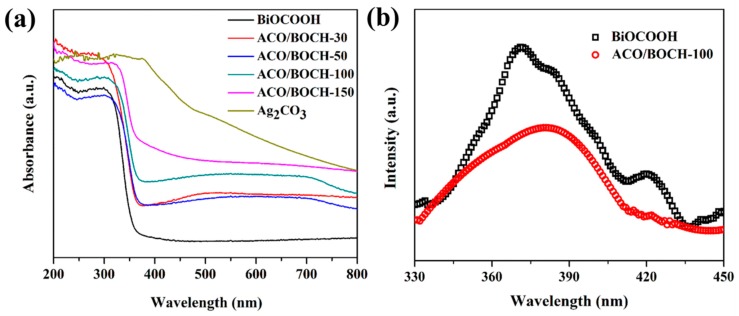
(**a**) UV-vis DRS of pure BiOCOOH, Ag_2_CO_3_, and Ag_2_CO_3_/BiOCOOH heterojunctions (ACO/BOCH-30, ACO/BOCH-50, ACO/BOCH-100, and ACO/BOCH-150). (**b**) PL spectra of pure BiOCOOH and ACO/BOCH-100 with an excitation wavelength of 300 nm.

The optical absorption behaviors of BiOCOOH, Ag_2_CO_3_, and Ag_2_CO_3_/BiOCOOH heterojunctions (ACO/BOCH-30, ACO/BOCH-50, ACO/BOCH-100, and ACO/BOCH-150) were studied by UV-vis diffuse reflectance spectrum (UV-vis DRS). As shown in Figure 4a, Ag_2_CO_3_ displayed intense absorbance in the VL region, whereas BiOCOOH had absorbance in the ultraviolet (UV) light region. The absorption band edges of pristine Ag_2_CO_3_ and BiOCOOH were about 470 and 365 nm, respectively, in agreement with previously reported results. The light absorption near the band edge followed the Tauc equation: α*hν* = A (*hν* − E_g_)*^n^*^/2^, where α, *h*, *ν*, and A were the absorption coefficient, Planck’s constant, light frequency, and the constant, respectively. According to the calculated Tauc’s plot (Figure S3), the band gaps (*E*_g_) of Ag_2_CO_3_ and BiOCOOH were 2.17 and 3.40 eV, in accordance with the results previously reported [8,23,32].

The conduction band (CB) and valence band (VB) potentials of BiOCOOH and Ag_2_CO_3_ were calculated by the empirical equations:

*E*_VB_ = *X* − *E*_0_ + 0.5*E*_g_ (1)

*E*_CB_ = *E*_VB_ − *E*_g_ (2)

where the *X* is the absolute electronegativity of the semiconductor, the *E*_0_ value equals to ~4.5 eV, *E*_g_ is the band gap of the semiconductor. Based on the equations above, the *E*_CB_ and *E*_VB_ of BiOCOOH were estimated to be −0.67 and 2.73 eV, and those of Ag_2_CO_3_ were calculated as 0.43 and 2.60 eV.

The photoluminescence (PL) was measured to investigate the separation efficiency of electrons and holes [36,37,38,39]. Figure 4b presents the PL spectra of BiOCOOH and ACO/BOCH-100. Clearly, BiOCOOH had a strong emission peak centered at around 370 nm. The PL emission peak of ACO/BOCH-100 was weaker than that of bare BiOCOOH, signifying that the recombination of carriers was pronouncedly reduced.

### 3.2. Photocatalytic Performance

**Figure 5:**
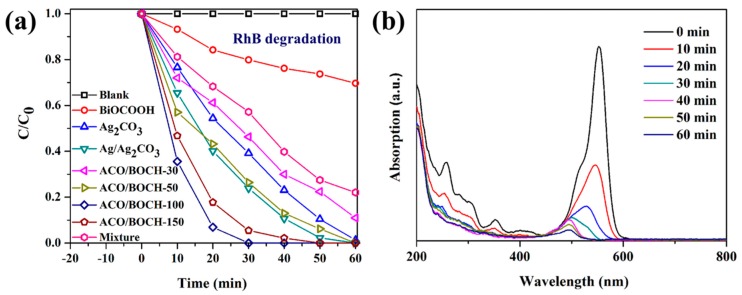
(**a**) Photocatalytic degradation of RhB dye with various catalysts under simulated sunlight. (**b**) Absorption spectra of RhB with irradiation time in the presence of ACO/BOCH-100.

The photocatalytic performances of Ag_2_CO_3_/BiOCOOH were assessed by degrading industrial dyes (RhB and MB), and antibiotic (TC) under simulated sunlight. Figure 5a presents the RhB degradation curves with various catalysts. No RhB was degraded in the absence of catalysts. The RhB degradation efficiency by using BiOCOOH, Ag_2_CO_3_ or Ag/Ag_2_CO_3_ reached 31.3%, 98.7% or 100% in 60 min of reaction. As BiOCOOH combined with Ag_2_CO_3_, the activity of these heterojunctions was pronouncedly enhanced and much higher than pure BiOCOOH. Among these Ag_2_CO_3_/BiOCOOH heterojunctions, ACO/BOCH-100 showed the highest photocatalytic activity with 100% of RhB degraded within 30 min, which was much higher than using Ag_2_CO_3_/Ag (76.1%), or Ag_2_CO_3_ (60.8%). To illustrate the synergistic effect between Ag_2_CO_3_ and BiOCOOH, the RhB photodegradation efficiency using the mechanical mixture (50 wt% Ag_2_CO_3_ + 50 wt% BiOCOOH) as the catalyst, was tested, which was much lower than that achieved by using ACO/BOCH-100, revealing that the formation of the Ag_2_CO_3_/BiOCOOH heterojunction can effectively boost the photocatalytic performance. Notably, the content of Ag_2_CO_3_ in the heterojunction played a significant role in enhancing the photocatalytic performance of as-prepared samples. However, when the loading amount of Ag_2_CO_3_ was high (ACO/BOCH-150), BiOCOOH was decorated by excessive Ag_2_CO_3_ nanoparticles with a relatively larger size, resulting in decreased photocatalytic activity.

Figure 5b shows the degradation curves of RhB with the reaction time in the presence of ACO/BOCH-100. Obviously, the major absorption peak at 554 nm rapidly decreased as the reaction time prolonged, indicating the decomposition of the structure. After 30 min of reaction, 100% of RhB was degraded, demonstrating the distinguished photocatalytic activity of ACO/BOCH-100 under simulated sunlight.

The reaction kinetics of RhB degradation over various catalysts was further studied. The experimental data could be fitted well with the pseudo-first-order model (Figure S4). It was found that ACO/BOCH-100 had the highest rate constant value of 0.1335 min^−1^, approximately 22.6, 2.1, or 5.2 folds that of pristine BiOCOOH (0.0059 min^−1^), Ag_2_CO_3_ (0.0638 min^−1^), or the mixture (0.0258 min^−1^).

**Figure 6:**
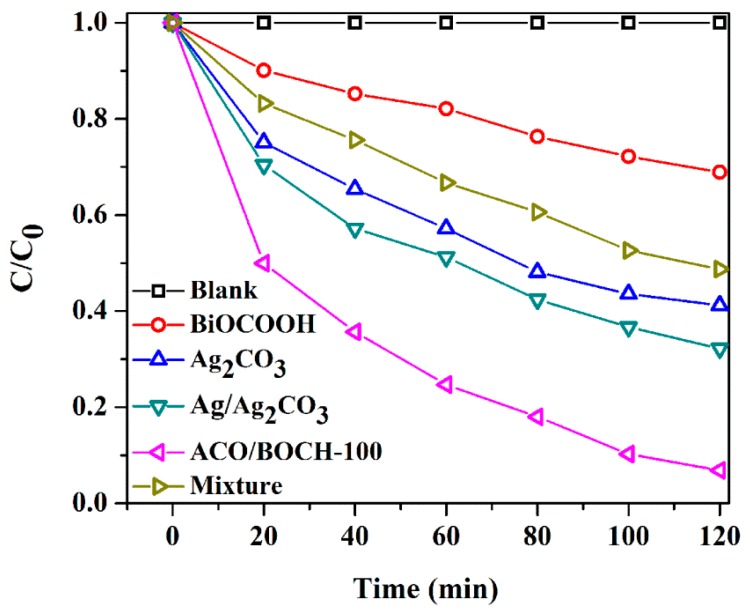
Photocatalytic degradation of TC with different catalysts under simulated sunlight.

MB dye and TC antibiotic, two refractory contaminants were also used to further investigate the photocatalytic activity of Ag_2_CO_3_/BiOCOOH under simulated sunlight (Figure 6, Figures S5 and S6). Notably, the MB degradation efficiency gained by employing ACO/BOCH-100 was as high as 100% within 60 min of irradiation, markedly higher than that gained by employing pristine BiOCOOH (42.7%), Ag_2_CO_3_ (79.3%), Ag/Ag_2_CO_3_ (89.7%), or a mechanical mixture (70.4%) as the photocatalyst. Additionally, a similar phenomenon was observed when using TC (Figure 6 and Figure S6) as a model contaminant. The optimal ACO/BOCH-100 possessed high photocatalytic activity with a TC degradation efficiency of 93.4% in 120 min, much greater than those of pure BiOCOOH (31.8%), Ag_2_CO_3_ (60.3%), Ag/Ag_2_CO_3_ (67.2%), as well as their physical mixture (52.3%). The results indicated that Ag_2_CO_3_/BiOCOOH had no-selectivity and could effectively decompose various toxic pollutants under simulated sunlight.

The photocatalytic performances of other reported catalysts for RhB removal were listed in Table S1. Notably, our catalyst showed a higher removal rate than other catalysts with similar/higher dosage of catalyst. Therefore, the Ag_2_CO_3_/BiOCOOH heterojunction possesses an excellent photocatalysis property for removing organic pollutants under simulated sunlight.

**Figure 7:**
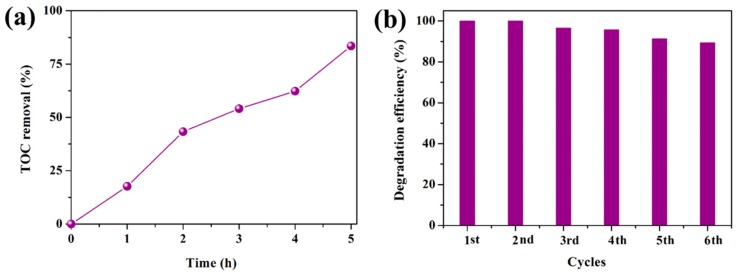
(**a**) TOC removal efficiencies over ACO/BOCH-100. (**b**) Cycling runs in the removal of RhB dye over ACO/BOCH-100.

The mineralization ability of a catalyst is vital for its practical application [40,41]. Therefore, the TOC values during the degradation of RhB (150 mL, 50 mg L^−1^) over ACO/BOCH-100 (200 mg) were determined. As shown in Figure 7a, 83.7% of TOC was removed after 5 h of irradiation, indicating that ACO/BOCH-100 holds a huge potential for the deep treatment of toxic contaminants.

The reusability of ACO/BOCH-100 was investigated via recycling for six runs in the removal of RhB and each run lasted 30 min. Figure 7b shows the photocatalytic performance of recycled ACO/BOCH-100. It was observed that the degradation efficiency of RhB at 30 min of the sixth run was 89.2%, which showed no apparent decrease compared with that (100%) of the first run, demonstrating the good stability of ACO/BOCH-100. During the photocatalytic process, it was found that there was a mass loss of 30 mg for the first run to 25.3 mg for the sixth run. Thus, the slight decline in activity should be attributed to the loss of catalysts during the recycling process. Moreover, the XRD characterization has demonstrated the formation of Ag_2_CO_3_/Ag after recycling experiments (Figure S7), which has been recognized as a stable hetero-structure [25,32]. These results indicate that ACO/BOCH-100 belongs to a type of stable catalysts.

### 3.3. Photocatalytic Mechanism

**Figure 8:**
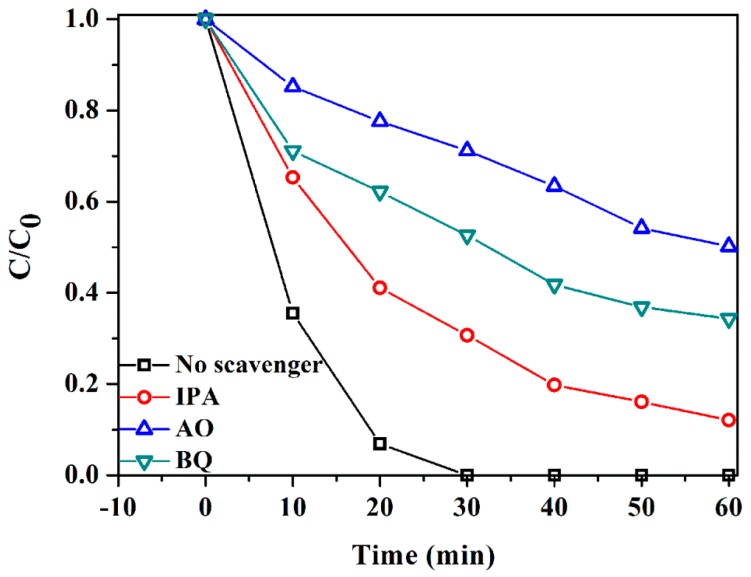
Radical–scavenge tests in the removal of RhB in the presence of ACO/BOCH-100.

Radical trapping experiments were conducted to investigate the effect of reactive species on the degradation of RhB over Ag_2_CO_3_/BiOCOOH [3,30,42]. As indicated in Figure 8, the RhB degradation efficiency reached 100% in the absence of a quencher. With the introduction of isopropanol (IPA), benzoquinone (BQ) and ammonium oxalate (AO), the photocatalytic activity of ACO/BOCH-100 was greatly suppressed, with RhB degradation efficiencies of 87.9%, 65.7% and 49.8%, respectively. The results demonstrated that •OH, •O_2_^−^ and h^+^ were generated during photocatalysis, and •O_2_^−^ and h^+^ were mainly responsible for RhB degradation over Ag_2_CO_3_/BiOCOOH.

**Figure 9:**
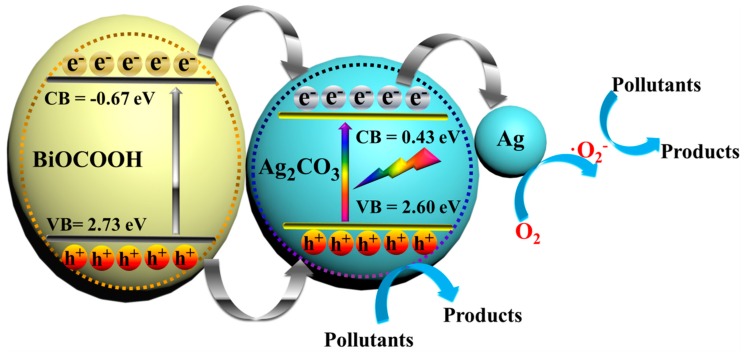
Proposed mechanism of pollutant removal over Ag_2_CO_3_/BiOCOOH under simulated sunlight.

On the basis of the results from systematic characterizations, the possible photocatalytic mechanism for contaminant degradation over Ag_2_CO_3_/BiOCOOH under simulated sunlight was proposed as outlined in Figure 9. Electrons and holes are produced in Ag_2_CO_3_ and BiOCOOH under simulated sunlight. Afterwards, the photo-generated electrons on CB and holes on VB of BiOCOOH tend to transfer to those of Ag_2_CO_3_ accordingly. Such a transfer process is beneficial to the separation of electrons and holes [43], as verified by the PL characterization (Figure 4b). Additionally, the photoelectrons can drift to the surface of Ag_2_CO_3_, leading to the formation of metallic Ag via the reduction of partial Ag^+^ (Ag_2_CO_3_). The produced metallic Ag can further facilitate electron migration to retard the recombination of charge carriers [44]. Finally, the electrons stored on the CB of metallic Ag can react with O_2_ to yield •O_2_^−^ radicals, which can be consumed to degrade toxic pollutants (RhB/MB/TC). Meanwhile, the holes that have accumulated on the VB of Ag_2_CO_3_ are involved in the degradation of toxic contaminants. Apparently, the results illustrate that the construction of type I heterojunction between Ag_2_CO_3_ and BiOCOOH can facilitate the separation of charge carriers, further improving the photocatalytic performance.

## 4. Conclusions

Ag_2_CO_3_ nanoparticles interspersed-BiOCOOH heterojunction photocatalytsts were prepared by a facile procedure. In comparison with pure Ag_2_CO_3_ and BiOCOOH, Ag_2_CO_3_/BiOCOOH (ACO/BOCH-100) exhibited superior photocatalytic activity for the degradation of toxic pollutants (RhB, MB, and TC) under simulated sunlight. The synergy effect between Ag_2_CO_3_ and BiOCOOH not only expanded the sunlight absorption range but also promoted the separation of electron and holes, leading to the outstanding performance of Ag_2_CO_3_/BiOCOOH. This novel photocatalyst can be employed to degrade refractory pollutants under simulated sunlight.

## Supplementary Materials

The following are available online at http://www.mdpi.com/2079-4991/8/11/914/s1, Figure S1: SEM image of pristine BiOCOOH, Figure S2: N_2_ adsorption-desorption isotherms of BiOCOOH and ACO/BOCH-100. The inset is the corresponding pore-size distributions, Figure S3: The Tauc plots of BiOCOOH and Ag_2_CO_3_, Figure S4: Pseudo-first-order kinetic plots and rate constants of RhB degradation over various photocatalysts, Figure S5: The MB degradation curves over different samples, Figure S6: Absorption spectra of TC with irradiation time in the presence of ACO/BOCH-100, Figure S7: The XRD patterns of ACO/BOCH-100 before and after cycling tests, Table S1: Summary of reported photocatalysts for degradation of RhB.
