# Supplementary material for: Ag2CO3 Decorating BiOCOOH Microspheres with Enhanced Full-Spectrum Photocatalytic Activity for the Degradation of Toxic Pollutants
Source: Nanomaterials (Basel). 2018 Nov 7;8(11):914. doi: 10.3390/nano8110914 (PMC6267195; doi:10.3390/nano8110914)
Supplement: Supplementary file 1 [file nanomaterials-08-00914-s001.pdf]

Ag_2CO_3 Decorating BiOCOOH Microspheres with Enhanced Full-Spectrum Photocatalytic Activity for the Degradation of Toxic Pollutants

Shijie Li[1,*], Liuye Mo[1], Yanping Liu[2,*], Yaming Ge[1], Huiqiu Zhang[1], and Yingtang Zhou[1]

[1] Key Laboratory of key technical factors in Zhejiang seafood health hazards, Institute of Innovation & Application, Zhejiang Ocean University, Zhoushan, Zhejiang Province, 316022, China. lishijie@zjou.edu.cn (S. L.); liuyemo@zjou.edu.cn (L. M.); geyaming@126.com (Y.G.); zhanghuiqiu2006@163.com (H. Z.).

[2] Department of Environmental Engineering, Zhejiang Ocean University, Zhoushan, Zhejiang Province, 316022, China; liuyp@zjou.edu.cn (Y. L.).

* Correspondence: lishijie@zjou.edu.cn (S. L); liuyp@zjou.edu.cn (Y. L.).

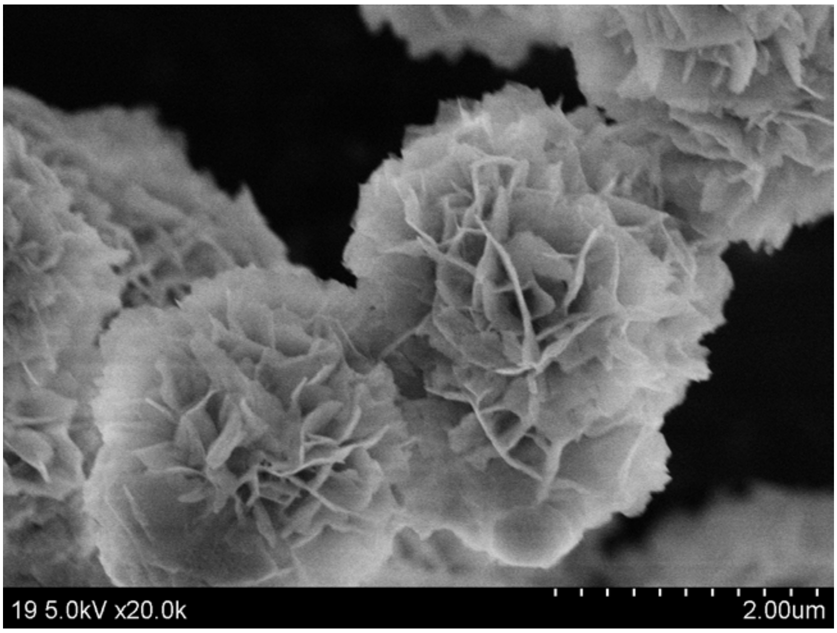

Figure S1. SEM image of pristine BiOCOOH.

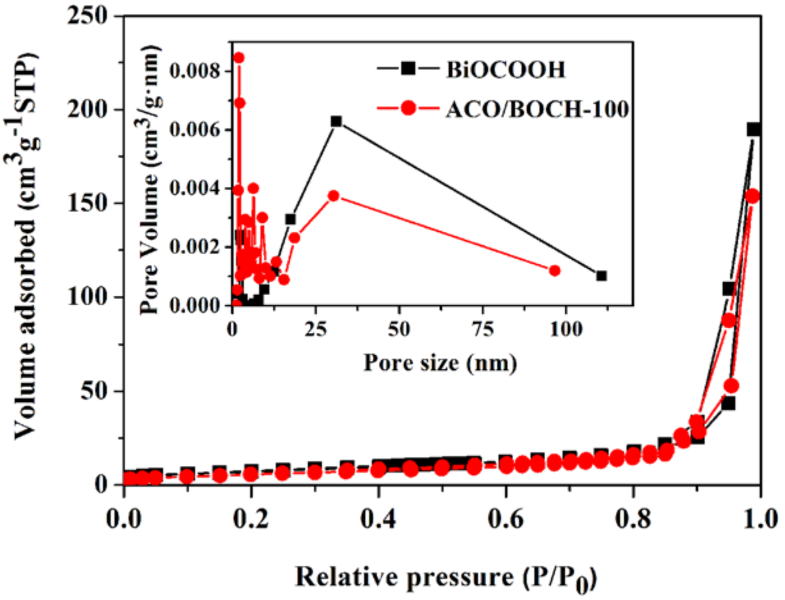

Figure S2. N_2 adsorption-desorption isotherms of BiOCOOH and ACO/BOCH-100. The inset is the corresponding pore-size distributions.

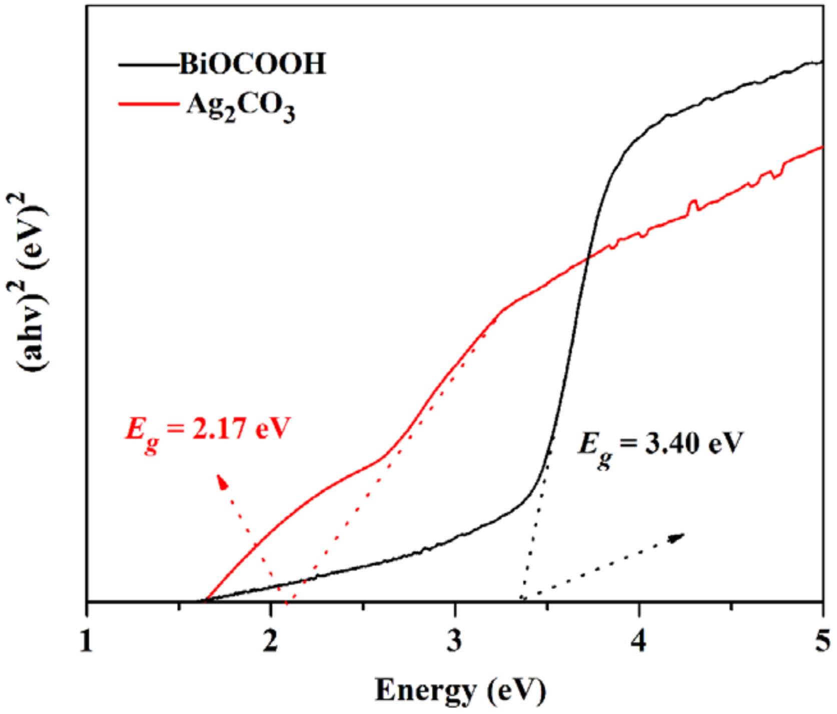

Figure S3. The Tauc plots of BiOCOOH and Ag_2CO_3

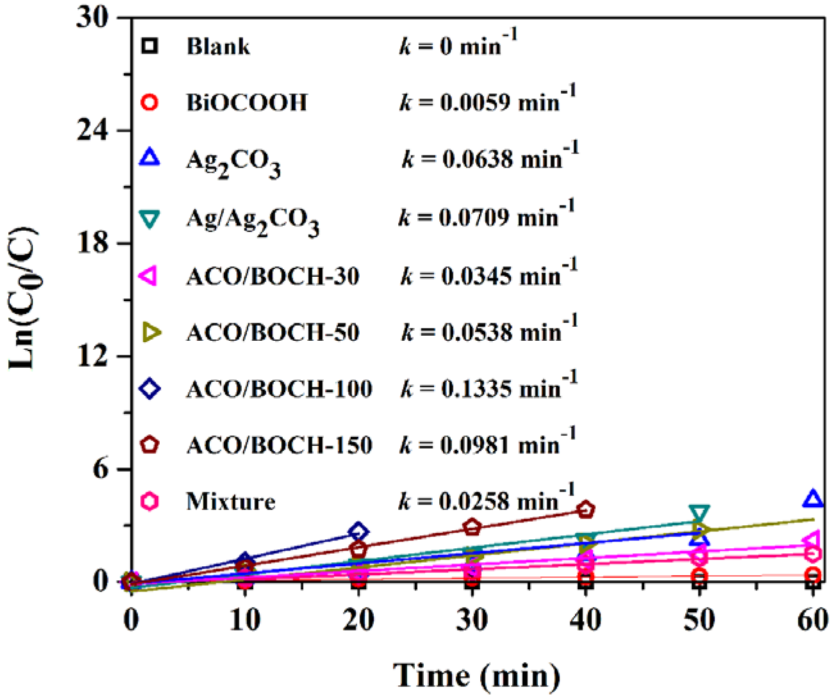

Figure S4. Pseudo-first-order kinetic plots and rate constants of RhB degradation over various photocatalysts.

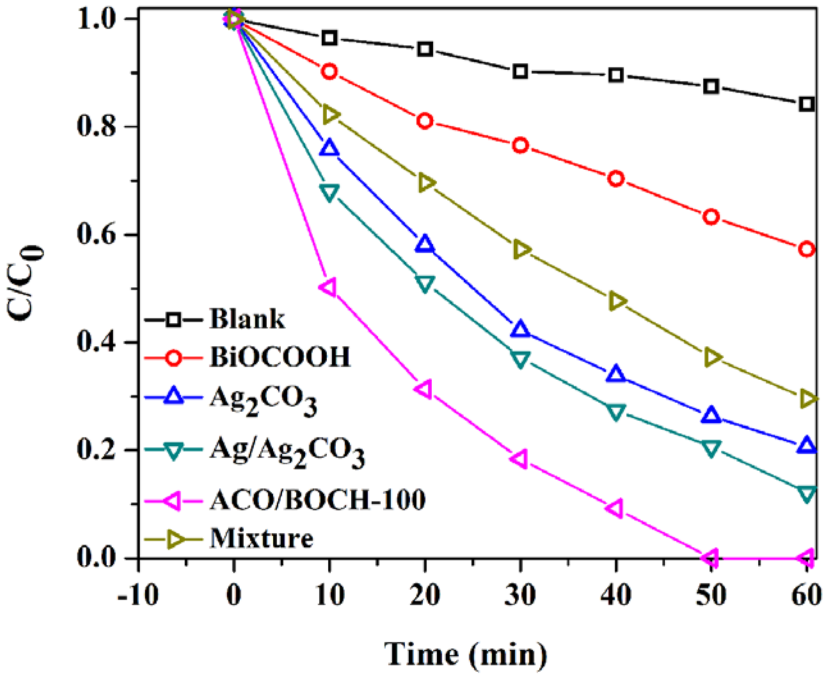

Figure S5. The MB degradation curves of different samples.

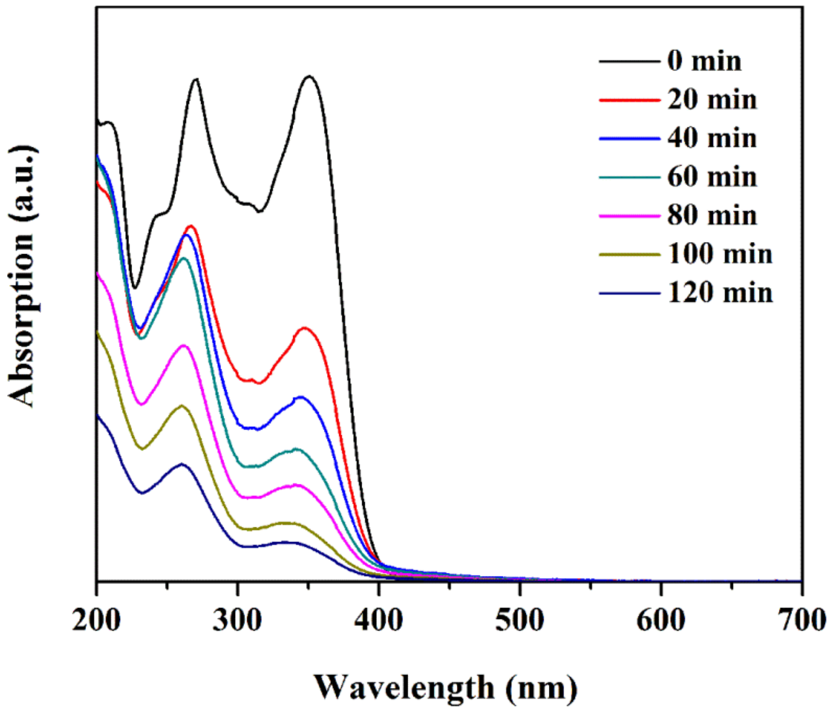

Figure S6. Absorption spectra of TC with irradiation time in the presence of ACO/BOCH-100.

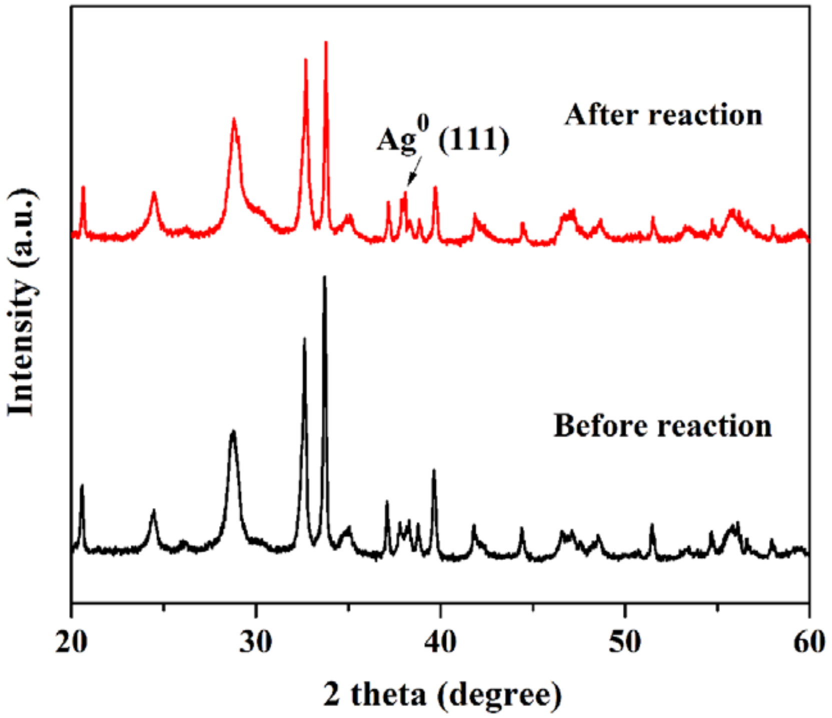

Figure S7. The XRD patterns of ACO/BOCH-100 before and after cycling tests.

Table 1. Summary of reported photocatalysts for degradation of RhB.

Photocatalyst	Dosage of sample	Concentration of RhB	Removal %	Light source	Time	Ref.
Ag_2CO_3/BiOCOOH	0.3 g/L	10 mg/L	100	simulated sunlight	30 min	This work
Ag_2CO_3/Ag/WO_3	0.5 g/L	20 mg/L	99.13	Visible light	60 min	32
F-Bi2MoO6	0.5 g/L	20 mg/L	78	simulated sunlight	100 min	41
MWCNTs/BiOCOOH	0.3 g/L	6 mg/L	92.1	simulated sunlight	60 min	20
Ag2CO3/Bi2O2CO3	1.0 g/L	10 mg/L	94	Visible light	120 min	26
AgI/BiOCOOH	0.3 g/L	10 mg/L	100	Visible light	60 min	17
